# Hydrocarbons Biodegradation by *Rhodococcus*: Assimilation of Hexadecane in Different Aggregate States

**DOI:** 10.3390/microorganisms10081594

**Published:** 2022-08-08

**Authors:** Luong Thi Mo, Puntus Irina, Suzina Natalia, Nechaeva Irina, Akhmetov Lenar, Filonov Andrey, Akatova Ekaterina, Alferov Sergey, Ponamoreva Olga

**Affiliations:** 1 Department of Biotechnology, Tula State University, Prospekt Lenina 92, 300012 Tula, Russia; 2 Russian-Vietnamese Tropical Research and Technology Center (Southern Branch), No. 1–3, 3 Thang 2 (the 3rd of February) Street, 11th Ward, District 10, Ho Chi Minh City 740500, Vietnam; 3 Laboratory of Plasmid Biology, Skryabin Institute of Biochemistry and Physiology of Microorganisms of Russian Academy of Sciences—A Separate Subdivision of Federal State Budget Institution of Science, Federal Research Centre, Pushchino Scientific Center of Biological Research of Russian Academy of Sciences, Prospekt Nauki 5, 142290 Pushchino, Russia; 4 Laboratory of Microbial Cytology, Skryabin Institute of Biochemistry and Physiology of Microorganisms of Russian Academy of Sciences—A Separate Subdivision of Federal State Budget Institution of Science, Federal Research Centre, Pushchino Scientific Center of Biological Research of Russian Academy of Sciences, Prospekt Nauki 5, 142290 Pushchino, Russia; 5 Laboratory of Ecological and Medical Biotechnology, Tula State University, Friedrich Engels Street 157, 300012 Tula, Russia

**Keywords:** hydrocarbon biodegradation, *Rhodococcus*, biosurfactants, solid and liquid substrate, hexadecane, adaptation, cell ultrastructure

## Abstract

The aim of our study was to reveal the peculiarities of the adaptation of rhodococci to hydrophobic hydrocarbon degradation at low temperatures when the substrate was in solid states. The ability of actinobacteria *Rhodococcus erythropolis* (strains X5 and S67) to degrade hexadecane at 10 °C (solid hydrophobic substrate) and 26 °C (liquid hydrophobic substrate) is described. Despite the solid state of the hydrophobic substrate at 10 °C, bacteria demonstrate a high level of its degradation (30–40%) within 18 days. For the first time, we show that specialized cellular structures are formed during the degradation of solid hexadecane by *Rhodococcus* at low temperatures: intracellular multimembrane structures and surface vesicles connected to the cell by fibers. The formation of specialized cellular structures when *Rhodococcus* bacteria are grown on solid hexadecane is an important adaptive trait, thereby contributing to the enlargement of a contact area between membrane-bound enzymes and a hydrophobic substrate.

## 1. Introduction

Actinobacteria of the genus *Rhodococcus* are widespread in nature and are isolated from aquatic and soil biotopes adjoining, in particular, oil and gas deposits. Due to a range of catabolic traits and unique enzyme systems, rhodococci have the ability to degrade hydrocarbon compounds representative of various chemical structures. These capabilities, including the ability of these bacteria to survive in unfavorable environmental conditions, make this genus suitable for the development of biopreparations for bioremediation of oil- and oil-products-contaminated sites [1,2,3,4].

Temperature is one of the major limiting factors significantly affecting bioremediation. In low-temperature environments, the bioavailability, solubility, and evaporation of oil hydrocarbons decrease and the viscosity of the oil increases. In this case, the diffusion processes and the level and rates of biodegradation of oil hydrocarbons by microorganisms slow down [5].

A low bioavailability of hydrocarbon substrates to microbial attack largely results from extremely poor solubility of these substrates in water. The factors due to which *Rhodococcus* strains, capable of utilizing hydrophobic substrates, assimilate hydrocarbons are as follows [6]: (1) the formation of the hydrophobic cell wall containing lipophilic compounds, thereby playing an essential role in establishing a direct contact of the cells with hydrocarbon drops, and (2) the release of biosurfactants, that can solubilize hydrocarbons in the aqueous phase, into the medium. The ability to produce biosurfactants is a common feature among microorganisms living in cold environments [7].

Rhodococci are shown to produce trehalolipid biosurfactants which are released into the cultivation medium by bacteria or bound to the bacterial cell wall. Mostly, nonionic trehalolipids (mono-, di-, tetra-, hexa- and octaacyl derivatives of trehalose) [8,9] and anionic trehalolipids (succinoyl trehalolipids) [10,11,12,13,14] are released into the cultivation medium. The information on trehalolipid biosurfactants produced by rhodococci was summarized in a recent review [15]. Glycolipids released into the cultivation medium can be replaced by phospholipids, thereby resulting in a decrease in the hydrophobic effect of the cell wall [16]. Thus, rhodococci can use the biosurfactants they produce to regulate cell surface properties in order to attach to or detach from hydrophobic substrates or hydrophobic surfaces [17,18,19]. This may also be a way for a more efficient transport of hydrophobic substrates into the cell. It was previously reported that the oil-degrading bacteria identified as *R. erythropolis S67* and *R. erythropolis X5* released succinoyl trehalolipids in the cultivation medium. The main components of these glycolipids were isolated and identified. They represented a mixture of isomeric homologues: 2,3,4-decanoyl-octanoyl-succinyl-2′-decanoyltrehalose, 2,3,4-dioctanoyl-succinyl-2′-decanoyltrehalose, 2,3,4-dioctanoyl-succinyl-2′-octanoyltrehalose, and 2,3,4-didecanoyl-succinyl-2′-decanoyltrehalose. It was shown that low temperature had no effect on the qualitative composition of trehalolipids produced by strain S67 [20].

It is noteworthy that the biodegradation of hydrocarbons is substantially dependent on their aggregate state, which can vary with temperature. For instance, n-hexadecane (in the coming text, we will call it hexadecane) can be found in liquid state at 26 °C, and solid state at 18 °C (melting point of hexadecane: 18.2 °C). In earlier reports [21,22], the degradation of hydrocarbons (tetradecane, kerosene, diesel fuel) was studied at low temperatures; nevertheless, the growth substrate was in a liquid aggregate state.

The aim of our study was to reveal the peculiarities of the adaptation of the oil-degrading bacteria *R. erythropolis* S67 and *R. erythropolis* X5 to hydrophobic hydrocarbon degradation at low temperatures when the substrate was in solid states using hexadecane as a model.

## 2. Materials and Methods

### 2.1. Microorganisms and Cultivation Conditions

The strains *R. erythropolis* X5 (VKM Ac-2532 D) and *R. erythropolis* S67 (VKM Ac-2533 D) were obtained from the collection of the Laboratory of Plasmid Biology, IBPM RAS. The biopreparation “MicroBak” contains these bacteria and is used for bioremediation of oil-contaminated areas [4]. The *R. erythropolis* X5 genome project has been deposited in the NCBI database under GenBank accession numbers CP044283 and CP044284, BioSample number SAMN12818508, BioProject number PRJNA573614, and SRA accession number PRJNA573614 [23].

For the cultivation of microorganisms, the Evans liquid synthetic minimal mineral medium and rich Luria–Bertani broth were used. The Evans medium had the following composition (L^−1^): K_2_HPO_4_, 8.71 g; 5 M solution NH_4_Cl, 1 mL; 0.1 M solution Na_2_SO_4_, 1 mL; 62 mM solution MgCl_2_,1 mL; 1 mM solution CaCl_2_, 1 mL; 0.005 m M solution (NH_4_)_6_Mo_7_O_24_, 1 mL; and trace elements solution, 1 mL; the pH was adjusted to 7.5 by the addition of concentrated HCl. The trace element solution in 1% HCl solution contained (g L^−1^): 0.41 ZnO, 2.9 FeCl_2_, 1.28 MnCl_2_, 0.13 CuCl_2_, 0.26 CoCl_2_, and 0.06 H_3_BO_3_. Glucose, hexadecane, naphthalene, crude oil, and diesel fuel were used as a sole carbon and energy source (2% v v^−1^ or w v^−1^). The Luria–Bertani broth had the following composition (L^−1^): bacto-tryptone (Difco, Detroit, MI, USA), 10 g; yeast extract (Difco, Detroit, MI, USA), 5 g; and NaCl, 10 g. To obtain agar media, bacterial agar (Difco, Detroit, MI, USA) was additionally added in the amount of 20 g L^−1^ of the medium. The prepared media were sterilized by autoclaving for 30 min at 120 °C (additional pressure: 0.5 atm). Microorganisms were cultivated in 750 mL Erlenmeyer flasks at 26 °C and 10 °C on an Excella 25 orbital shaker (Eppendorf, Hamburg, Germany) at 180 rpm. The microbial growth was monitored by optical density measurements at 600 nm (OD_600_) using a spectrophotometer Cintra 6 (GBC Scientific Equipment Pty. Ltd., Melbourne, Australia).

### 2.2. Measuring Surface Tension

The surface tension was measured on a surface tensiometer (Surface Tensiomat model 21; Fisher Scientific, Cole-Parmer, United States) at 25 °C. The surface tension of the reference solution (Evans medium) was 77 mN m^−1^.

### 2.3. Measurement of the Content of Glycolipid Biosurfactants

The content of the biosurfactant was assessed by measuring the concentration of the carbohydrate part of the glycolipid with a photocolorimetric method (Petrikov et al., 2013). Cells were preliminarily removed from the cultivation medium by centrifugation at 10,000 rpm and 4 °C for 10 min on a Rotanta 460R centrifuge (Hettich-Zentrifugen, Tuttlingen, Germany). A 2 mL sample of the supernatant was placed into a test tube and 20 μL of 80% phenol solution in water was added. With vigorous stirring, 5 mL of concentrated sulfuric acid (ρ 1.84 g mL^−1^) was added and left for 10 min. Then, the solution was vigorously stirred again, and the test tube was placed in the water bath at 25 °C for 20 min. The optical density of the resulting solution was measured on a UV–visible spectrophotometer (Cintra 6, GBC Scientific Equipment Pty. Ltd., Melbourne, Australia), using a quartz cell with a 1 cm path length. Wavelengths in the range of 480–490 nm were chosen as optimum wavelengths for estimating the given carbohydrate by the absorption maximum. The reference solution was prepared in a similar way, using distilled water instead of the sample. The concentration of carbohydrate part was determined by using previously created calibration curves. To calculate the concentration of the biosurfactant, the obtained value of the sugar concentration was then multiplied by a factor of 2.5, equal to the ratio of the molecular weight of the glycolipid (862) to the molecular weight of the appropriate carbohydrate (342 for trehalose).

### 2.4. Hydrophobicity of the Cell Surface of Microorganisms

The hydrophobicity was measured by the MATH test (microbial adherence to hydrocarbon) (Rosenberg 1984) as modified by Satpute et al. (2010). The cultivation medium was centrifuged using a TG16-WS tabletop high speed centrifuge (Polikom, Russia) at 10,000× *g* for 10 min. The cell biomass was washed with distilled water twice and resuspended in a phosphate-magnesium buffer containing (g L^−1^): K_2_HPO_4_·3H_2_O, 22.2 g; KH_2_PO_4_, 7.26 g; urea, 1.8 g; and MgSO_4_·7H_2_O, 0.2 g; the pH was 7.0. In this case, the OD_600_ of the cell suspension of all samples should be in the range of 0.48–0.50. An amount of 0.5 mL of n-hexadecane was added to 3.0 mL of the cell suspension. The contents of the tubes were shaken vigorously on a shaker at 2000 rpm for 3 min. After being left to stand at room temperature for 10 min, the separation of the aqueous and hydrocarbon phases occurred. The hydrocarbon phase was carefully separated from the aqueous phase and the OD_600_ of the aqueous phase was measured. The hydrophobicity was calculated using the formula:

$$H\left(\%\right)=\left(1-\frac{A_{0}}{A}\right)\times 100,$$

where *H* (%) was the hydrophobicity of the cell surface (%); *A*_0_, *A* were the values of the OD_600_ of the cell suspension before and after n-hexadecane addition, respectively.

### 2.5. Transmission Electron Microscopy

Bacterial cells were fixed in a 1.5% solution of glutaraldehyde in 0.05 M cacodylate buffer (pH 7.2) at 4 °C for 1 h, washed three times with the same buffer and additionally fixed in a 1% solution of OsO_4_ in 0.05 M cacodylate buffer (pH 7.2) at 20 °C for 3 h. After dehydration, the material was embedded in epoxy resin Epon-812. Ultrathin sections were mounted on the supporting mesh grids and contrasted for 30 min in a 3% uranyl acetate solution in 70% alcohol and additionally stained with lead citrate.

From a sample of cells grown in a medium with hexadecane, we obtained five repeats for the preparation and analysis of ultrathin cell slices. In each repeat, we analyzed 5–7 slices. We observed 15–20 cells on each slice.

### 2.6. Total Cell Lipid Content in Bacteria

The total cell lipids were extracted with polar organic solvents from raw bacterial biomass after the excess of hexadecane was removed by using hexane. The raw biomass (40–50 mg of cells per dry weight) was diluted with water to a volume of 1 mL. The suspension was placed in a centrifuge tube with a ground-in glass stopper, and 3.75 mL of chloroform–methanol (1:2, v:v) was added; the mixture was shaken and left at room temperature for several hours with intermittent shaking. The cells were precipitated by centrifugation, and the extract was decanted into another 15 mL centrifuge tube. Microbial cells were resuspended in 4.75 mL of chloroform–methanol–water (1:2:0.8, v:v:v), and the mixture was then shaken and centrifuged. To the combined supernatant, 5 mL of chloroform: methanol (1:1, v:v) was added; the chloroform layer was separated by centrifugation. The lower chloroform layer was diluted with benzene and evaporated to dryness in vacuum on a rotary evaporator (30–35 °C). The lipid residue was immediately dissolved in chloroform–methanol (1: 1, v v^−1^), the solution was then subjected to centrifugation and brought up to the required volume with chloroform.

### 2.7. Content of Cell-Bound Hexadecane

To remove excess n-hexadecane from the outer surface of cells, 5–10 mL of a mixture of ethanol–butanol–chloroform (10:10:1, v:v:v) was added to 1 g of biomass and resuspended. Then, the suspension was centrifuged for 10 min at 10,000× *g*. Washing was done twice. To the resulting precipitate, 5–10 mL of 5 M NaOH was added and left overnight; after that, the reaction mixture was extracted with dichloromethane twice. The extract was dried over anhydrous sodium sulfate, the solvents were then evaporated in a rotary evaporator at 40 °C and a pressure of 0.2 atm to dryness. The content of cell-bound hexadecane was analyzed by gas chromatography [24].

### 2.8. Determination of Fatty Acid Composition of Lipids

The fatty acid composition of lipids was determined by gas chromatography with a mass spectrometric detector (GC–MS). For this, the transesterification of fatty acid esters with methanol was carried out according to the procedure in [12] with modification. A weighed sample (30 mg for lipids) was resuspended in 2 mL of a mixture of concentrated sulfuric acid–methanol (5% by volume) and heated at 60 °C for 2 h. Then, after cooling, it was extracted with 5 mL of diethyl ether. The residue of sulfuric acid in the extract was washed with distilled water to neutral pH. Further, the stripped-off sample was dissolved in chloroform and analyzed using an Agilent 6890N chromatograph (Agilent Technologies Inc., Santa Clara, CA, USA) with an Agilent 5973 mass spectrometric detector on an HP-1 capillary column (30 m × 25 mm × 25 µm) with a temperature gradient from 50 to 290 °C for 40 °C per min, then held for 30 min at 290 °C. Helium was used as a carrier gas. Fatty acids were identified using the NIST/EPA/NIH database (http://www.nist.gov/srd/nist1a.cfm (accessed on 13 July 2022)).

### 2.9. Determination of Residual Hexadecane in the Cultivation Medium

Hexadecane was extracted from the culture broth with an equal volume of hexane. Using a microsyringe, 10 µL^−1^ of the sample was injected into the evaporator and analyzed for the hexadecane content using a Chrystal-5000 gas chromatograph (Khromatek-Analytic, Yoshkar-Ola, Russia) with a BP1J08 capillary column (30 m × 0.3 mm × 0.53 µm). The flow rate of the carrier gas (nitrogen) was 174.6 mL min^−1^. The column temperature was set at 250 °C, the evaporator temperature was 300 °C, and the detector temperature was 250 °C. At least two chromatograms of the analyzed samples were recorded. The content of hexadecane in the samples was determined using a calibration graph, which expressed the dependence of the peak area on the content of n-hexadecane. The degree of degradation of hexadecane was calculated using the following formula:

$$D\left(\%\right)=\frac{C_{1}-C_{0}}{C_{0}}\times 100,$$

where *C*_1_ was the concentration of hexadecane in the liquid cultivation medium after the cultivation of microorganisms (% of the volume of the medium), and *C*_0_ was the concentration of hexadecane in sterile control.

### 2.10. Experimental and Statistics Analysis

Results obtained were analyzed using the built-in statistical packages in Excel (MS Office 2007). All experiments were performed in three replicates. In each experiment, we took samples for the chemical and biochemical analyses. Each sample was measured three times.

## 3. Results

### 3.1. Production of Biosurfactants by Bacterial Strains X5 and S67 of R. erythropolis during Degradation of Hexadecane in Different States of Aggregation

The cultivation of strains X5 and S67 was carried out in a mineral medium with liquid hexadecane at 26 °C and solid hexadecane at 10 °C. The surface tension was 27 mN m^−1^ when both strains were grown on medium containing hexadecane at 26 °C, and during bacterial growth at 10 °C, the surface tension was 32 mN m^−1^ and 42 mN m^−1^ for X5 and S67, respectively, indicating the abilities of these bacteria to produce biosurfactants even at low temperatures.

During the cultivation of strain X5 on hexadecane at 26 °C, an emulsion appeared in the medium, and this emulsion was distributed evenly throughout the volume, and its turbidity increased with further cultivation for 6 days, while during the cultivation of strain S67, a film formed on the surface of the medium after 24 h; the suspension in the medium volume was less dense than that of strain X5. During the cultivation of S67 in a medium containing hexadecane, the pH of the liquid cultivation medium decreased from 7 to 4 (at the end of the experiment).

**Figure 1:**
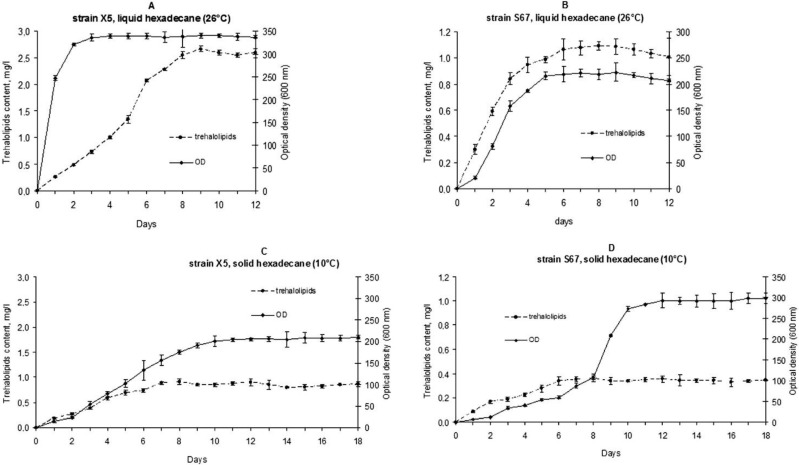
The kinetics of cell growth and trehalolipids contents for strains X5 and S67 during batch cultivation in Evans medium with hexadecane at 26 °C (**A**,**B**) and 10 °C (**C**,**D**).

At 26 °C, the maximum optical density of the liquid culture medium (OD_600_ 2.9) for strain X5 was higher than that for S67 (OD_600_ 0.8) (Figure 1A,B). This can be explained by the formation of a suspension in the culture medium for *R. erythropolis* X5 and the formation of a biofilm on the surface of the medium for *R. erythropolis* S67. The optical density and number of *R. erythropolis* S67 cells in the liquid cultivation medium were low, since the majority of *R. erythropolis* S67 cells were located in the surface film. The content of glycolipids in the cultivation medium in the stationary growth phase for both strains was ~270–300 mg L^−1^ (Figure 1A,B).

During the cultivation of bacteria in the mineral medium at 10 °C (Figure 1C,D), when hexadecane was in a solid state of aggregation, a gradual formation of a suspension was observed throughout the volume of the medium for *R. erythropolis* X5 and a thin film at the border with solid hexadecane for *R. erythropolis* S67. Along with the growth of bacteria on hexadecane, the content of trehalolipids in the culture medium in the stationary phase increased, whose the maximum value reached ~100 mg L^−1^ for both strains (Figure 1C,D).

The assessment of the degree of degradation of hexadecane by the studied strains at two temperatures was carried out taking into account the particular growth phases. The studied strains S67 and X5 were shown to degrade hexadecane most efficiently in the exponential growth phase at both temperatures (more than half of the total utilized substrate) (Figure S1). The degrees of liquid hexadecane biodegradation (at 26 °C) in the exponential growth phase were 34–35%, and at 10 °C in a medium with solid hexadecane, the rates of hexadecane consumption were 27–28%. In the stationary growth phase, strain X5 utilized 53% of hexadecane during cultivation at 26 °C (8 days) and 40% at 10 °C (18 days). Strain S67 in the same phase utilized 46% and 30% of hexadecane at 26 °C and 10 °C, respectively. It should be noted that at 10 °C, despite the solid state of aggregation of hexadecane, the rate of its degradation remained high: 40% for *R. erythropolis* X5 and 30% for *R. erythropolis* S67.

### 3.2. Hydrophobicity of the Cell Wall of R. erythropolis Strains X5 and S67 during Growth on Hexadecane in Different Aggregated States

**Figure 2:**
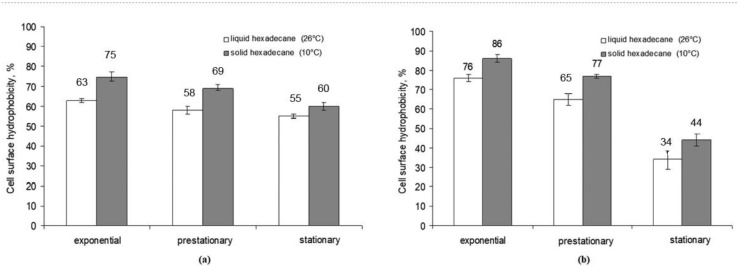
Cell wall hydrophobicity of *R. erythropolis* strains X5 (**a**) and S67 (**b**) during batch cultivation with hexadecane in different aggregate states.

To reveal how the aggregate state of the hydrocarbon affected the change in cell surface hydrophobicity of *Rhodococcus* strains X5 and S67, the adhesive activity to hexadecane of cells grown on a medium with liquid and solid hexadecane was determined. Samples were taken at particular phases of cell growth. The cell surface hydrophobicity of both strains was higher when cultured in a medium with solid hexadecane (75% for X5 and 86% for S67) compared to liquid hexadecane (63 and 76%, respectively) (Figure 2). It should be noted that the cell wall hydrophobicity of both *Rhodococcus* strains varied depending on the growth phase. In the exponential growth phase, it was maximum and then decreased in the stationary phase.

### 3.3. Isolation and Characterization of Total Cell Lipids of Bacterial Strains X5 and S67, Grown on Medium with Hexadecane in Different Aggregation States

To reveal the role of lipids in the adaptation of rhodococci in the consumption of hydrocarbons in different aggregate states, the content and fatty acid composition of lipids in cells of strains X5 and S67, grown on a mineral medium with liquid and solid hexadecane, were determined. The content of total cell lipids (TCL) was significantly higher in the cells of the studied strains X5 and S67, grown on a medium with solid hexadecane (Figure S2). The maximum values were 168 and 115 mg g^−1^ of biomass for strain X5, 143 and 92 mg g^−1^ of biomass for strain S67 in the exponential growth phase on solid and liquid hexadecane, respectively.

To determine the content of fatty acids in the total cell lipids of bacteria, the methanolysis of lipids isolated from the biomass was carried out. The total mass of methyl esters of fatty acids and their composition were determined by gas chromatography, and the total content of fatty acids was calculated based on their ratios in the mixture. Gas chromatographic analysis of lipid extracts of cells of *Rhodococcus* strains X5 and S67 showed insignificant amounts (0.006–0.05% of the total content of cellular lipids) of hexadecane (Table 1).

The main component in the total composition of fatty acids was hexadecanoic acid, an intermediate of hexadecane degradation (36% at 26 °C and 31% at 10 °C for strain X5; 52% at 26 °C and 33% at 10 °C for strain S67) (Table 2). However, strains X5 and S67 differed in the fatty acid composition of their cellular lipids: saturated branched acids were present in cells of strain X5 grown on liquid hexadecane and saturated unbranched fatty acids were found in cells of strain S67 (Table 2). Being cultured on solid hexadecane, the cells of both strains changed the ratio of saturated unbranched and branched fatty acids, and the proportion of unsaturated 9-hexadecenoic acid in X5 cells increased (33%) and saturated branched fatty acids appeared in S67 cells (37%).

### 3.4. Extracellular Lipids from Cultivation Medium of Strains X5 and S67 Grown on Medium with Hexadecane in Different Aggregate States

To study the fatty acid composition of extracellular lipids, the culture liquid was separated from the cells grown on medium with hexadecane at different temperatures and it was further analyzed by GC/MS after transesterification with methanol. The lipid extracts of the cell-free culture medium contained a number of fatty acids with chain lengths from C_8_ to C_16_ (Figures S3 and S4). Extracts contained hexadecanoic acid (about 25% for strain S67, 5% for strain X5), which is the first fatty acid in the degradation pathway of hexadecane by rhodococci cells. Interestingly, decanoic acid was the predominant fatty acid in lipid extracts at both 26 °C and 10 °C. Since the degradation products of hexadecane are fatty acids, the presence of fatty acids in the cultivation medium indicates that the catabolic pathway of hexadecane starts already in the pericellular area.

### 3.5. Morphology and Ultrastructural Organization of Cells of R. erythropolis Strains X5 and S67 When Grown on Hexadecane in Different Aggregate States

Light microscopy (phase contrast) revealed different positions of X5 and S67 cells in relation to hexadecane in cultivation medium at 10 °C. X5 cells were located at the hexadecane–water interface (outside the hydrophobic drop), while S67 isolates were seen inside the hydrophobic drop (data not shown).

**Figure 3:**
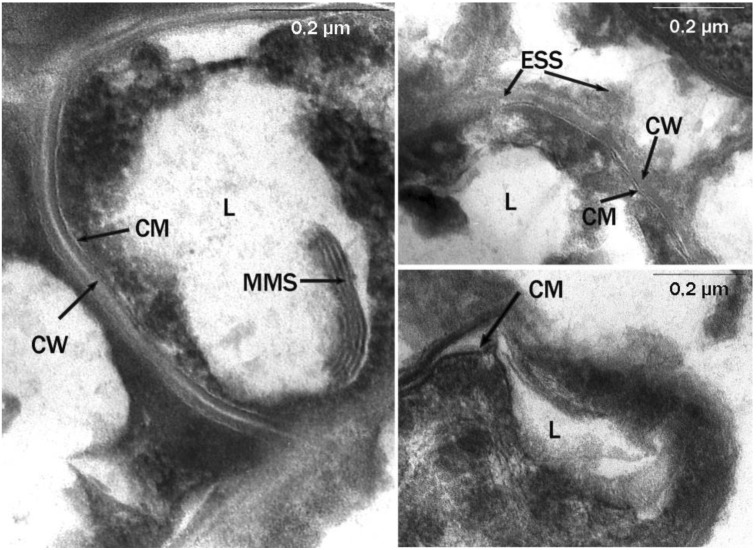
Ultrathin sections of *R. erythropolis* S67 cells (cultivation conditions: liquid hexadecane, 26 °C; 6 days). Intracytoplasmic membrane structures of various morphologies are visible. CW, cell wall; MMS, multimembrane structures; CM, cytoplasmic membrane; L, lipid inclusions; ESS, extracellular surface structures; 0.2-micron scale.

Transmission electron microscopy observation of ultrathin sections of X5 and S67 cells showed morphological differences in the cells of microorganisms grown on liquid and solid hexadecane. In microphotographs of the studied actinobacteria, grown on liquid hexadecane, the cytoplasmic membrane (CM) had an electron-transparent layer, and the cell wall (CW), typical of the ultrastructure for Gram-positive bacteria, was adjacent to the CM from the outside (Figure 3). It had the form of a dense homogeneous layer with a thickness of 20–30 nm. The outer surface of the cell wall was covered with a thin microfibrillar layer and various surface and extracellular surface ultrastructures (ESS) with a characteristic granular-fibrillar organization, which had a high affinity for ruthenium red. In the cytoplasm, there were electron-transparent inclusions (lipids, L), characteristic of substances of a hydrophobic nature, as a rule, with single, large, often polygonal in shape with sharply defined sharp corners and contours.

Multimembrane structures (MMS) were also found, mainly in the form of multiple, parallel membrane-like and loop-like or tubular formations, usually associated with hydrophobic inclusions (Figure 3). The MMS profile consisted of several electron-dense layers (analogous to hydrophilic layers of biological membranes), alternating with electron-transparent layers (analogous to hydrophobic zones of biological membranes). MMS were poorly developed inside the cytoplasm and were clearly visible only in the area of the limiting contour of inclusions.

**Figure 4:**
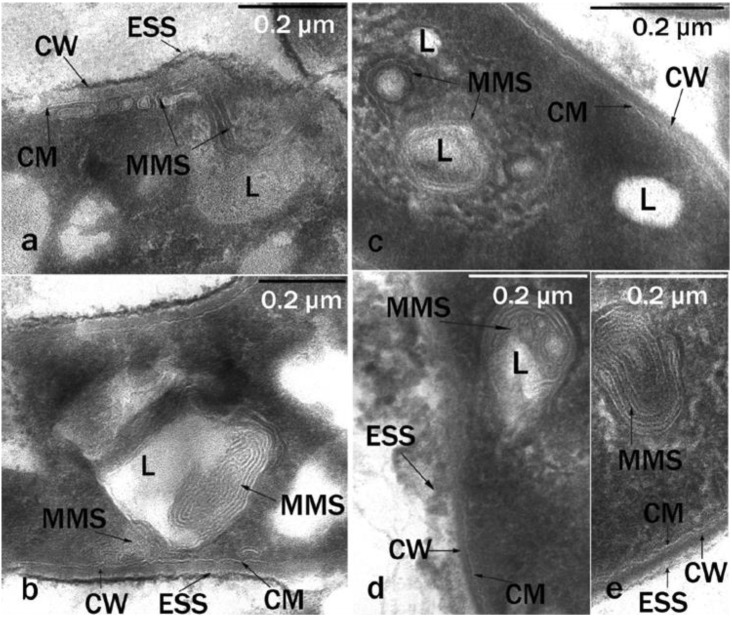
Ultrathin cell sections (cultivation conditions: solid hexadecane; 10 °C; 6 days): (**a**,**b**)—*R. erythropolis* S67; (**c**–**e**)—*R. erythropolis* X5. Intracytoplasmic membrane-like structures of various packaging are visible. CW, cell wall; MMS, multimembrane structures; CM, cytoplasmic membrane; L, lipid inclusions; ESS, extracellular surface structures; 0.2-micron scale.

Microbial cells grown on solid hexadecane differed significantly in their ultrathin organization from cells grown on liquid hexadecane. We were dealing with a pronounced adaptive ultrastructural rearrangement of cells, which allowed the cell to interact with a solid hydrophobic substrate, because it required, during the first stages of degradation, a preliminary change of the substrate into a more accessible form for transport into the cell. MMS were well-developed inside the cytoplasm, the lipid inclusions had a spherical shape, were of small or medium size, they were visible inside the cells, and the number of inclusions per cell section ranged from 1 to 3–4 (Figure 4). The inclusions were associated with numerous MMS, which indicated the role of these structures in the transport and primary transformation of solid hydrocarbons.

**Figure 5:**
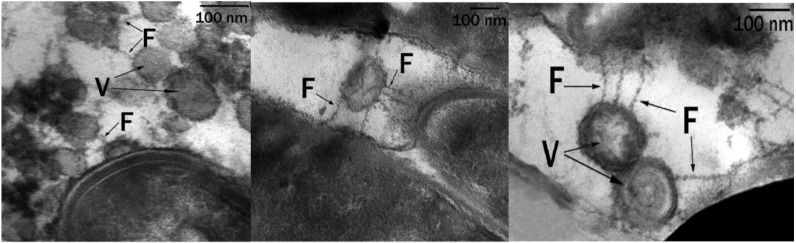
Ultrathin sections of *R. erythropolis* S67 cells (cultivation conditions: solid hexadecane, 10 °C, 6 days): Vesicles (V) are visible, surrounded by a shell of electron-dense layers, connected to bacterial cells by electron-dense fibers (F); 100 nm scale.

Near the surface and directly on the surface of the cells, multiple spherical structures with homogeneous contents were detected. These were vesicles (V) surrounded by a three-layer membrane (Figure 5). The outer surface of these formations was covered with a thin, loose layer with a high electron density. Electron-dense structures can appear as a result of the interaction of negatively charged succinoyl trehalolipids with positively charged ruthenium red. On ultrathin sections, electron-dense filaments (fibers, F) surrounded and connected the vesicles to the cell surface. These fibers were hollow pili-like structures (Figure 5, on the right).

In all cases we observed MMS in each cell in the field of view. Vesicles and fibers were detected only in samples of rhodococci grown in the presence of solid hexadecane at low temperature.

## 4. Discussion

It was previously shown that biosurfactants of rhodococci during degradation of hydrocarbons can be produced in a liquid cultivation medium or be bound to the cell wall. A number of works are devoted to the study of the production of biosurfactants by rhodococci at different temperatures [11,12,25]. Malavenda et al. [21] demonstrated that arctic rhodococci, when grown on tetradecane (melting point, 5.8 °C) at 15 °C, produced biosurfactants which could reduce the culture–medium surface tension in the range between 66 and 27 mN m^−1^. Other authors showed that when marine bacteria *Rhodococcus* sp. LF13 and LF22 grew on kerosene (melting point, −47 °C) under low temperature conditions (13 °C), the surface tension of the medium decreased to 37 and 30 mN m^−1^, respectively [22]. The surface tension of liquid cultivation medium of *Rhodococcus* sp. Q15 grown on diesel fuel (melting point, −55 °C) at 5 °C dropped to 40 mN m^−1^ [25]. However, it should be noted that in these studies, all the studied hydrophobic hydrocarbons were in a liquid state under a low temperature.

We compared the growth and consumption rates of the model substrate—hexadecane–by *R. erythropolis* strains X5 and S67 at 26 °C, when the substrate was liquid, and at 10 °C, when it was solid (melting point of hexadecane, 18.2 °C). The degradation of hexadecane was shown to occur most intensively in the exponential growth phase, when bacteria had consumed more than half of the total substrate. In 8 days, X5 consumed 53% of hexadecane in liquid state and 40% of solid hexadecane in 18 days. Despite the solid aggregate state of hexadecane, the degree of its degradation remained high: in the case of strain X5 it was 40% and 30% for strain S67 within 18 days. Earlier, Whyte et al. [26] showed *Rhodococcus* sp. Q15 was capable of utilizing about 30% of hexadecane after 30 days at 5 °C. When studying the biodegradation of hydrophobic substrates at a low temperature [27], *R. cercidiphyllus* strain BZ22 was found to consume 35% of hexadecane at 10 °C after a 14-day cultivation. These results are comparable to the results obtained in our experiments.

The transport of hydrocarbons by rhodococci can be divided into two stages. The first stage is a direct contact of the cells to the substrate suggesting that high cell surface hydrophobicity plays an essential role in the attachment of rhodococci, especially when the substrate is in a solid aggregated state. The second stage is that, when dissolved in membrane lipids, alkanes can be oxidized by membrane-localized enzymes to the corresponding carboxylic acids [10].

It is known that microorganisms can change cell surface hydrophobicity using biosurfactants [17]. The study showed that the cell surface hydrophobicity of strains X5 and S67 was higher when cells grew on solid hexadecane: thus, in the exponential growth phase, it was maximum and achieved 75% for X5 and 88% for S67. When cells grew on liquid hexadecane, the cell surface hydrophobicity was 63% and 76%, respectively. It should be noted that both strains revealed the highest cell surface hydrophobicity in the exponential growth phase and a decrease in cell surface hydrophobicity was detected in the stationary phase. A comparison of the degree of cell surface hydrophobicity and the content of extracellular biosurfactants indicated a common tendency toward a decreasing hydrophobicity of the cell wall with an increasing quantity of biosurfactants produced into the cultivation medium.

Margesin et al. [27] proposed that the external lipid barrier of permeability for hydrophobic substrates into actinobacteria cells is formed by high-molecular hydroxy acids—mycolic acids and their esters with trehalose, promoting the penetration of hydrocarbons into the cell in a passive-diffuse way.

In our experiments, the hydrophobicity of the bacterial cell wall decreased towards the stationary growth phase (Figure 2), and the degree of adhesion to hydrocarbons decreased. A decrease in the degree of cell surface hydrophobicity can be explained by the entry of succinoyl trehalolipids into the cultivation medium; this fact is consistent with the data of Franzetti et al. [28] on the production of biosurfactants by bacteria outside the cells. They showed [28] *Gordonia* cells in the early exponential growth phase had a high hydrophobicity of the cell surface due to cell-associated biosurfactants and remained attached to large drops of hydrophobic substrate, so the absorption of substrate was carried out by cells through direct contact. During the late exponential growth phase, the cells released bioemulsifiers into the culture medium and became more hydrophilic, which allowed them to attach to the hydrophilic outer layer of solubilized hydrocarbon droplets [28].

Factors involved in the cell surface hydrophobicity do not only include the presence of glycolipids in the cell wall. Psychrotrophic rhodococci cells may adapt to grow at low temperature by increasing the lipid content and reducing their degree of saturation [29]. Due to the ability of *Rhodococcus* strains to change the fatty acid composition of membrane lipids, they change the fluidity and permeability of the cell wall [30]. In our experiments, the growth of *R. erythropolis* strains X5 and S67 on solid hexadecane increased the content of total cell lipids. Strains X5 and S67 varied in the fatty acid composition of cellular lipids. Cells of strain X5 grown on liquid hexadecane contained saturated branched acids, while cells of strain S67 contained more saturated unbranched acids. When cultured on solid hexadecane, a change in the ratio of saturated unbranched and branched fatty acids was observed in the cells of both strains (Table 2). The proportion of unsaturated 9-hexadecenoic acid in the cells of strain X5 increased (33%). Saturated branched fatty acids appeared in the cells of strain S67 (37%) (Table 2). Apparently, strains X5 and S67 develop different mechanisms by which the cell membrane fluidity changes.

The analysis of cell lipids of the studied strains showed that the main component was hexadecanoic acid, an intermediate product of hexadecane degradation when cells were grown on both liquid and solid hexadecane (36% at 26 °C and 31% at 10 °C for strain X5; 52% at 26 °C and 33% at 10 °C for strain S67) (Table 2). Since the gas chromatographic analysis of lipid extracts from X5 and S67 cells showed insignificant amounts of hexadecane in the cells, this may indicate that hexadecane degradation gets started in the pericellular area.

The GC/MS method showed that a number of fatty acids with a chain length from C_8_ to C_16_ were present in the supernatant of the culture medium of strains X5 and S67 grown on hexadecane (Figures S3 and S4). It should be noted that in lipid extracts of the supernatant of the culture medium of strains X5 and S67, grown on both liquid at 26 °C and solid hexadecane at 10 °C, fatty acids with an even number of atoms were found. Under the action of the terminal alkane monooxygenase of aerobic bacteria, hexadecane is known to be oxidized to alcohol, which by alcohol dehydrogenase is further converted to aldehyde, and then aldehyde dehydrogenase converts hexadecane into acid. Further oxidation proceeds along the *beta*-oxidation of long-chain fatty acids, during which the fatty acid decreases by two carbon atoms. The presence of decanoic (C_10_) and octanoic (C_8_) acids may indicate not only hexadecane degradation, but also a possible synthesis of trehalolipids with the corresponding fatty acid “tails”. Peng et al. [31] studied lipids in the cell-free culture medium during the growth of *R. erythropolis* 3C-9 on hexadecane and found that the content of hexadecanoic acid (C_16_) was about 65%. Moreover, the presence of hexadecanoic acid in cell-free supernatant in our experiments confirmed that the catabolic transformation of hexadecane started in the pericellular area.

The study of the morphology and ultrastructural organization of cells of *R. erythropolis* strains X5 and S67, revealed multimembrane structures (MMS) on ultrathin sections, mainly in the form of multiple loop-like or tubular formations associated with hydrophobic inclusions (Figure 3 and Figure 4). The MMS profile consisted of several electron-dense layers alternating with electron-transparent layers, which was an analog of the hydrophilic and hydrophobic zones of biological membranes. MMS were more pronounced in cells grown on solid hexadecane (Figure 4). Similar structures were previously found in *Rhodococcus opacus* grown on benzoate [32]. The formation of multimembrane structures in the studied bacteria is an adaptive mechanism for their survival under extreme environmental conditions. The data of the electron cytochemical analysis of *R. erythropolis* strains confirmed the connection between the increase of membrane ultrastructures into the cells and the destructive processes of n-alkanes in the cold, when the hydrophobic substrates were solid. The MMS were involved in the transport of a hydrophobic substrate into the cell, as well as in the partial oxidation of the substrate under the action of membrane-localized enzymes [32].

Multimembrane structures (MMS) associated with inclusions are characteristic of cells during the degradation of hydrophobic and toxic compounds. Similar structures were previously found in *Sulfobacillus* during the oxidation of hydrophobic sulfur [33], in *Pseudomonas* capable of utilizing sodium dodecyl sulfate [34,35], in *Acinetobacter* sp. grown on hydrocarbons [36], and in rhodococci grown on liquid and gaseous n-alkanes [37].

In the cytoplasm, there were electron-transparent inclusions (L), typical for substances of a hydrophobic nature. Currently, there is no clear opinion about the composition of lipid inclusions formed in cells of actinobacteria, when they are grown on hydrophobic substrates. These inclusions can be hexadecane [38] or triacylglycerols (TAG) [39,40]. The information on the *rhodococci* ability to accumulate TAG when grown on various hydrophobic substrates has been summarized in the recent review [41]. The composition of the inclusions can be suggested by the content of cell-bound hexadecane and fatty acids in cellular lipids. Since cell lipids of strains X5 and S67 during growth on hexadecane were found to contain a low content of hexadecane and a high content of fatty acids (Table 1), we assumed that these electron-transparent intracellular inclusions were triacylglycerols.

The *Rhodococcus* X5 and S67 cells formed multiple spherical structures (vesicles, V), that could be regarded as an analog of liposomes (Figure 5). Spherical structures were formed by a type of self-assembly with the participation of polar molecules with hydrophobic and hydrophilic tails (biosurfactants) secreted by the cell. It is possible that these membrane-like structures were formed by the trehalolipid biosurfactants produced by our strains, X5 and S67. These structures may contain primary alkane degradation enzymes also secreted by cells. This form of encapsulation of primary biodegradation enzymes in the liposome is most effective for interaction with the solid substrate.

Similar structures named outer membrane vesicles (OMVs) were found for *Pseudomonas putida* KT2440 during cultivation in lignin-rich media [42]. OMVs contained many enzymes acting toward lignin-derived aromatic compounds. Salvachua et al. [42] hypothesized that some of the proteins could be trafficked to the extracellular compartment via OMVs. Lignin, like hexadecane at low temperature, is a solid substrate. The formation of numerous vesicles by rhodococcal cells during growth on solid hexadecane is also an important adaptive property that provides an increase in the contact area of bacterial cells with a hydrophobic substrate.

The transmission electron microscopy observation of ultrathin sections of X5 cells grown on solid hexadecane showed that vesicles were connected to cells by means of electron-dense filaments—fibers (F) (Figure 5). Delegan et al. [43] studied the ultrastructure of rhodococci cells grown on dodecane at 28 °C. They described surface fibrillar structures, which are most commonly used by bacterial cells to attach to the surface. In contrast, we showed that fibers were pili-like structures that were hollow and possibly served to transport hexadecane and its intermediates.

In our previous study, it was shown that *R. erythropolis* S67 cells released succinoyl trehalolipids into the culture medium [20]. Importantly, negatively charged succinoyl trehalolipids, which include a residue of succinic acid, may exhibit a high affinity for positively charged ruthenium red. This may lead to the appearance of electron-dense structures in the region of their localization. We suggest that extracellular biosurfactants (trehalolipids) are probably involved in the functioning of these channels.

**Figure 6:**
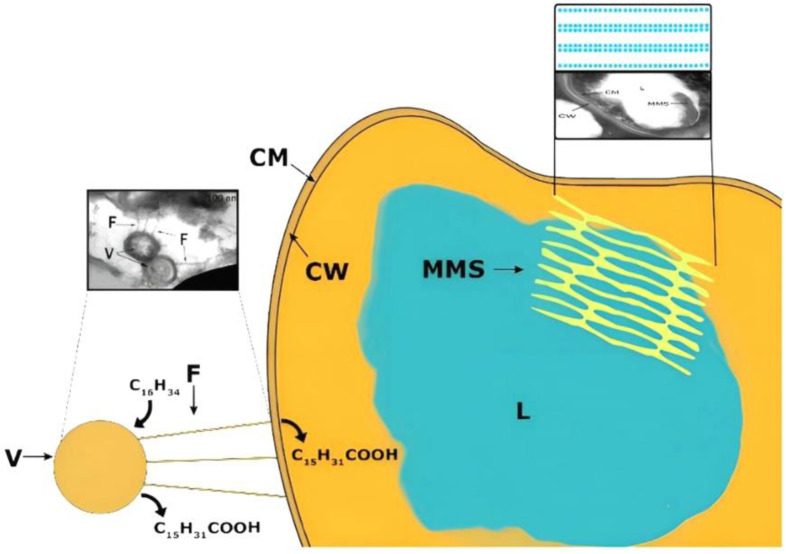
Mechanisms of hexadecane transformation by *Rhodococcus* bacteria.

The degradation of hexadecane (a hydrophobic hydrocarbon) by rhodococci in the liquid and solid aggregate states is realized by several mechanisms (Figure 6).

The utilization of liquid hydrocarbon by rhodococcal cells occurs mainly due to the production of biosurfactants in the cultivation medium and the formation of micelles that transport hexadecane to the cell. Further on the cell surface, hexadecane degradation begins under the action of membrane-bound enzymes, as evidenced by the appearance of degradation products in the medium (hexadecanoic and other fatty acids).

During the degradation of solid hydrocarbons, the hydrophobicity of the cell wall increases due to: (1) the formation of cell-bound biosurfactants; (2) the increase of the content of fatty acids in the cell wall; (3) the change in the composition of lipids in the cell wall. This study showed for the first time that during the degradation of solid hydrocarbons (namely, hexadecane) new cellular structures appeared: intracellular membrane structures and surface vesicles connected to the cell by electron-dense fibers. Electron-transparent inclusions, probably triacylglycerols, were observed in the cytoplasm. We suppose that vesicular structures play a key role in the degradation of solid hydrocarbons. The degradation of hexadecane gets started with the attachment of vesicles to it and its subsequent passive transport into these structures, where the partial degradation of hexadecane occurs due to the action of the primary enzymes of hydrocarbon oxidation. Further, the products of hydrocarbon degradation are transported to the cell through hydrophobic channels (fibers) with the participation of biosurfactants.

As we know, psychrotolerant microorganisms are extraordinary sources of new biomolecules with unique properties, which include biosurfactants. Biosurfactants play key ecological roles in many cold habitats of microbiota. They participate in carbon utilization processes by enhancing the bioavailability of poorly soluble compounds, including pollutants, in cold soils and marine environments. These properties can be used to develop new biotechnological products and processes in cold climates.

## 5. Conclusions

The formation of specialized cellular structures when *Rhodococcus* bacteria are grown on solid hexadecane is an important adaptive trait thereby contributing to the enlargement of a contact area between membrane-bound enzymes and a hydrophobic substrate. Understanding the mechanisms of biodegradation of hydrocarbons at low temperatures will expand our understanding of fundamental aspects of the microbial metabolism of the hydrophobic recalcitrant chemicals at extreme conditions and contribute to a feasible bioremediation technology in the future.

## Figures and Tables

**Table 1:** Content of lipids, fatty acids and hexadecane in bacterial biomass (mg g^−1^).

Lipid Components, mg g^−1^	*R. erythropolis* X5		*R. erythropolis* S67	
	26 °C	10 °C	26 °C	10 °C
TCL content	115 ± 12	168 ± 13	92 ± 7	143 ± 15
Methyl esters of fatty acids	81 ± 5	118 ± 12	66 ± 5	105 ± 8
Total content of fatty acids	76 ± 6	111 ± 9	62 ± 7	99 ± 10
Hexadecane	0.01 ± 0.01	0.05 ± 0.01	0.05 ± 0.01	0.14 ± 0.01

**Table 2:** Fatty acid composition of cell lipids of strains X5 and S67 when grown on hexadecane in different aggregate states.

Fatty Acids, %	*R. erythropolis* X5		*R. erythropolis* S67	
	26 °C	10 °C	26 °C	10 °C
Saturated unbranched				
Octanoic	-	3.7 ± 0.3	-	
Decanoic	-	8.2 ± 0.8	-	6.2 ± 0.4
Dodecanoic	5.4 ± 0.4	3.8 ± 0.6	5.2 ± 0.4	5.6 ± 0.8
Tetradecanoic	13.7 ± 1.0	9.4 ± 0.7	14.8 ± 1.0	-
Pentadecanoic	10.6 ± 1.0	-		
Hexadecanoic	35.6 ± 4.6	31.2 ± 2.9	51.8 ± 5.7	33.6 ± 2.4
Heptadecanoic			8.7 ± 0.6	
Total	65	56	81	45
Saturated branched				
12-methyl-tridecanoic			-	10.6 ± 0.4
14-methyl-hexadecanoic			-	14.2 ± 0.7
15-methyl-hexadecanoic	12.1 ± 0.7	11.6 ± 1.0		
4-4-dimethyl-hexadecanoic			-	12.3 ± 0.7
Total	12	11	0	37
Unsaturated				
9-hexadecenoic	22.7 ± 2	32.8 ± 3.1	19.4 ± 2.2	17.6 ± 4.2

## Data Availability

Data available from public libraries/open sources according to the references.

## Supplementary Materials

The following supporting information can be downloaded at: https://www.mdpi.com/article/10.3390/microorganisms10081594/s1, Figure S1: Hexadecane degradation by R. erythropolis strains X5 and S67 at 26 °C (a) and 10 °C (b) during batch cultivation in liquid Evans medium, Figure S2: Total cell lipids content in X5 (a) and S67 (b) strains during batch cultivation with hexadecane, Figure S3: Chromatograms of fatty acid methyl esters present in extracellular biosurfactants produced by strain X5 when grown on hexadecane in different aggregate states, Figure S4: Chromatograms of methyl esters of fatty acids present in extracellular biosurfactants produced by strain S67 when grown on hexadecane in different aggregate states.
