# Supplementary material for: Hydrocarbons Biodegradation by Rhodococcus: Assimilation of Hexadecane in Different Aggregate States
Source: Microorganisms. 2022 Aug 8;10(8):1594. doi: 10.3390/microorganisms10081594 (PMC9416576; doi:10.3390/microorganisms10081594)
Supplement: Supplementary file 1 [file microorganisms-10-01594-s001.zip › microorganisms-1841100-supplementary.pdf]

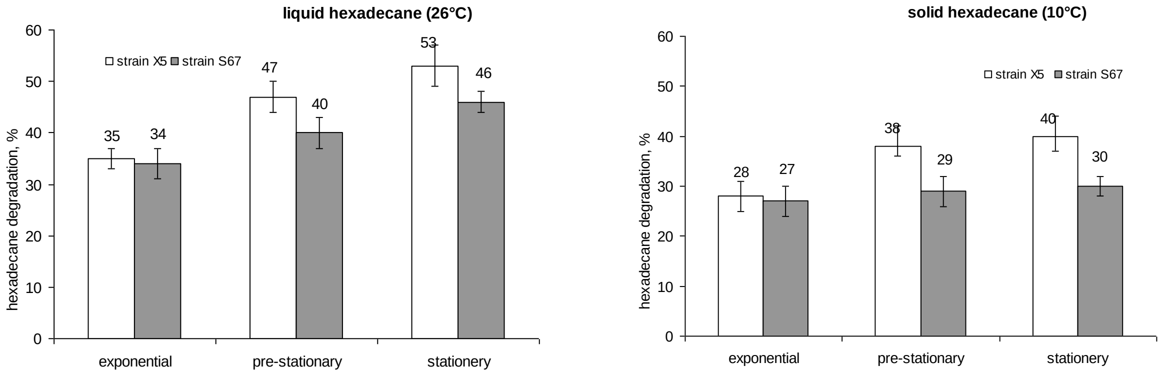

(a) (b)

Figure S1. Hexadecane degradation by *R. erythropolis* strains X5 and S67 at 26°C (a) and 10°C (b) during batch cultivation in liquid Evans medium.

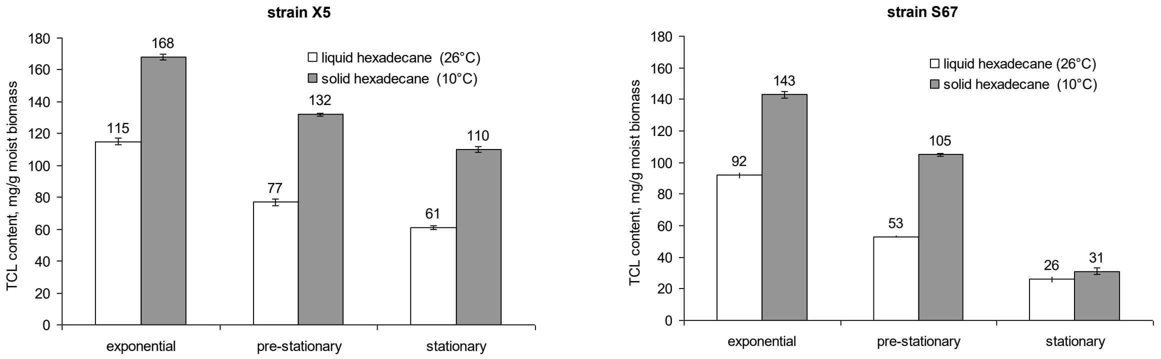

(a) (b)

Figure S2. Total cell lipids content in X5 (a) and S67 (b) strains during batch cultivation with [illegible]

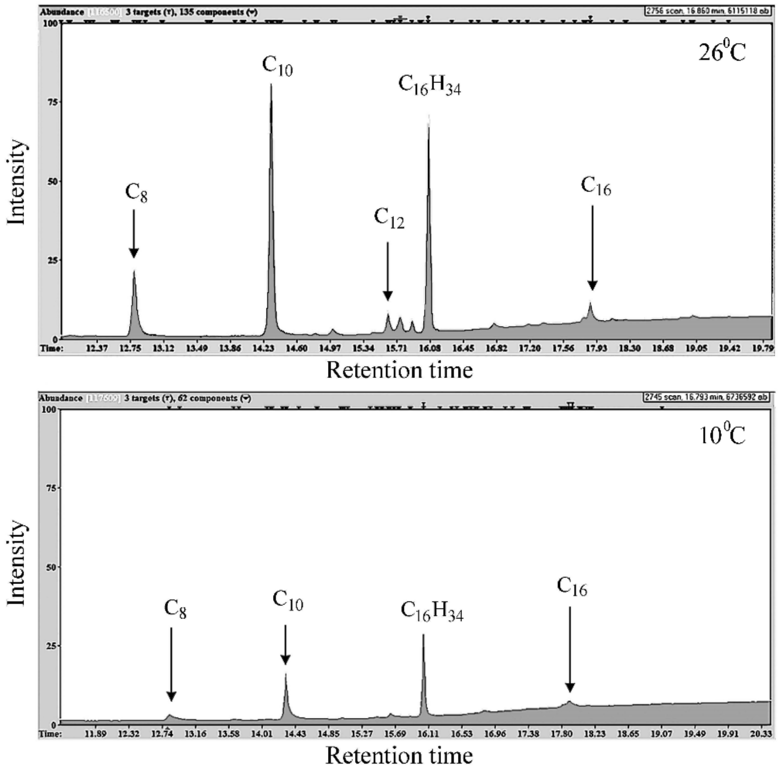

Figure S3. Chromatograms of fatty acid methyl esters present in extracellular biosurfactants produced by strain X5 when grown on hexadecane in different aggregate states.

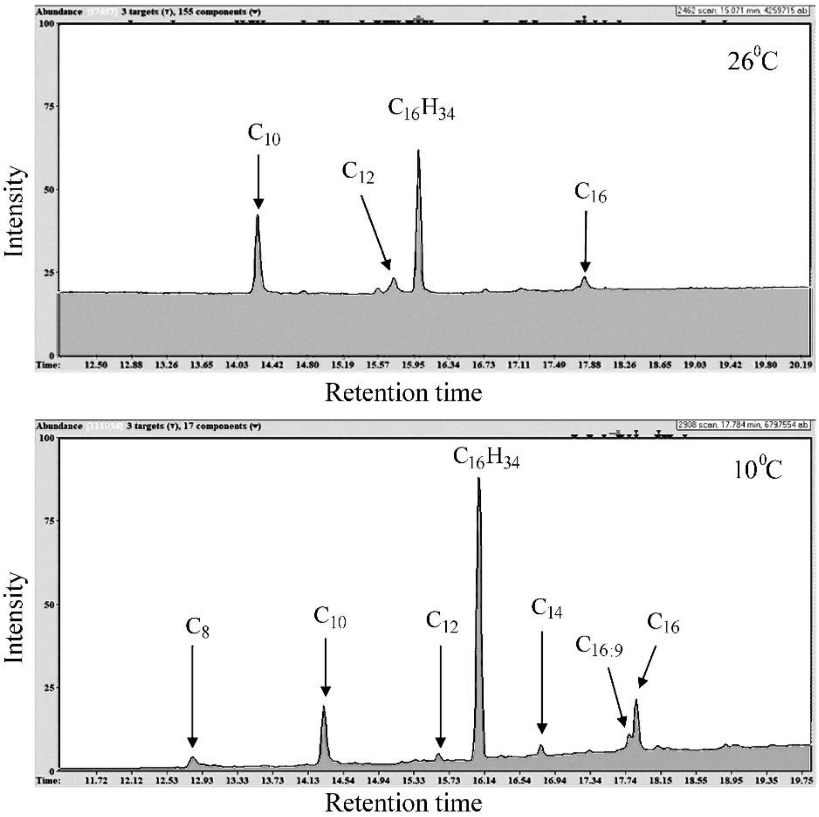

Figure S4. Chromatograms of methyl esters of fatty acids present in extracellular biosurfactants produced by strain S67 when grown on hexadecane in different aggregate states.
